# Supplementary figures and images for: Deciphering the interplay between biology and physics with a finite element method-implemented vertex organoid model: A tool for the mechanical analysis of cell behavior on a spherical organoid shell
Source: PLoS Comput Biol. 2025 Jan 10;21(1):e1012681. doi: 10.1371/journal.pcbi.1012681 (PMC11771887; doi:10.1371/journal.pcbi.1012681)

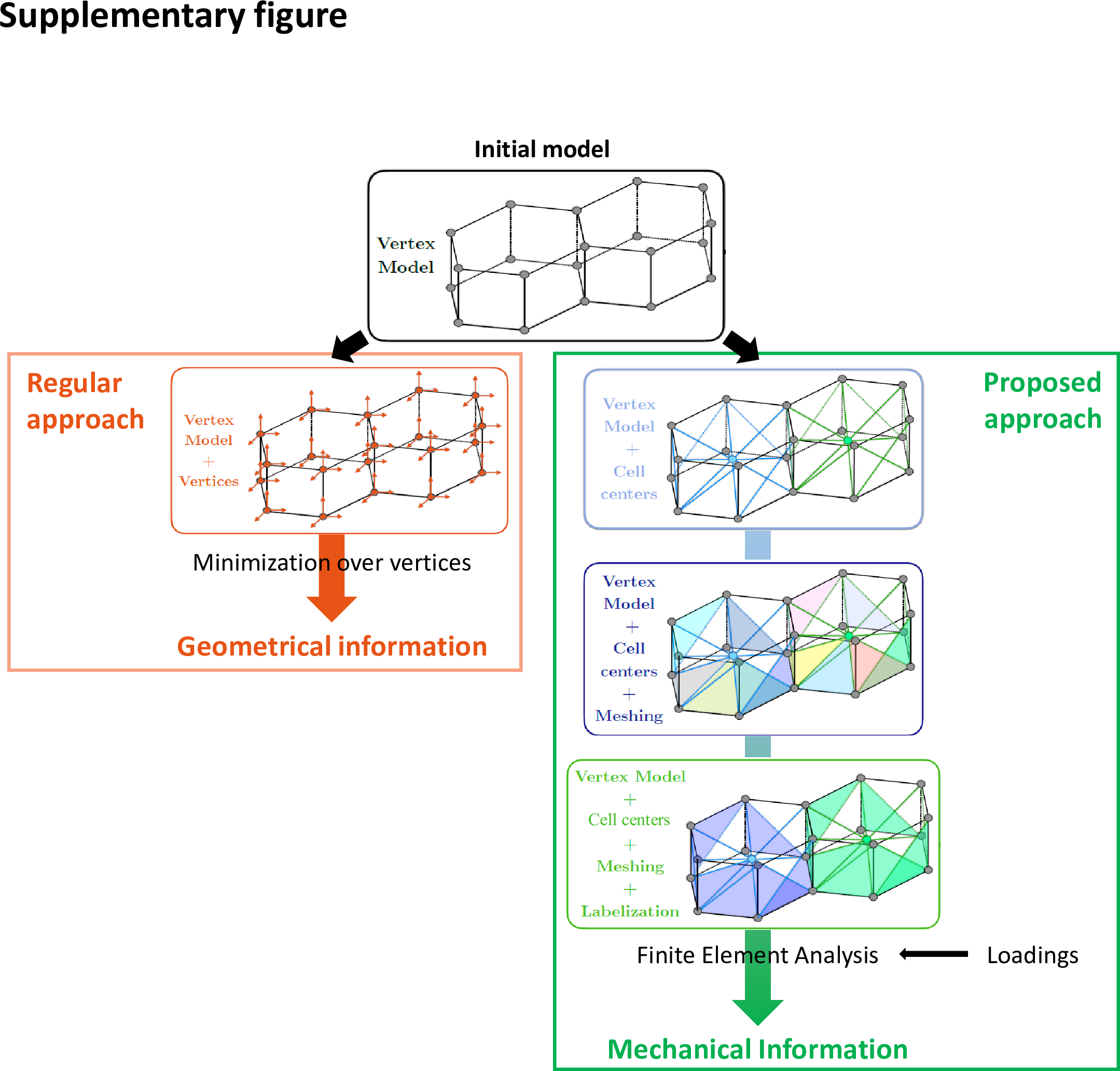

Supplement: S1 Fig — The vertex model uses the vertices of the mesh as optimization variables, thereby minimizing the energy of the active 3D vertex model to obtain geometric information. The proposed method uses these vertices as the initial geometric configuration, then meshes them by using finite elements, and finally labels them to decipher the individual cells. This approach enables geometric and mechanical information regarding not only points but also volumes to be obtained. (TIF) [file pcbi.1012681.s001.tif]
