# Supplementary material for: Deciphering the interplay between biology and physics with a finite element method-implemented vertex organoid model: A tool for the mechanical analysis of cell behavior on a spherical organoid shell
Source: PLoS Comput Biol. 2025 Jan 10;21(1):e1012681. doi: 10.1371/journal.pcbi.1012681 (PMC11771887; doi:10.1371/journal.pcbi.1012681)
Supplement: Attachment — Submitted filename: Responses to Reviewers.docx [file pcbi.1012681.s002.docx]

**Point-by-point responses to the review comments and description of the changes made on the manuscript PCOMPBIOL-D-24-00672 by Laussu et al.**

We appreciate that the first reviewer (R1) acknowledges that ‘*The question of time-dependent 3D deformations of tissue is important in physical biology’.* He highlights that our *‘work makes good computational progress and is validated by experiments’,* and that *‘It shows how the material deformations and constraints not captured by vertex modeling, can in fact be captured by including finite element description of material analysis’*.

We appreciate the reviewers' constructive criticism. We believe that our responses to the various points raised, the changes made in the revised manuscript and/or the additional information and figures we have attached to this letter will answer all their criticisms. It is important to note that we have edited the text to take account of all the referees' comments and suggestions. In the revised version of the manuscript, we have also modified the text to clarify the referees' concerns. Regarding English, the manuscript has been proofread and edited by a professional translator.

We do hope that our manuscript will now reach the standard for publication in *PLOS Computational Biology*.

### Response to Reviewer #1 (R1):

R1 raises few weaknesses that we all addresses either in this document or directly within the main text of the manuscript as specified here.

#### Major comments:

***• 1:*** *One of the advantages of finite element modeling is that it can handle large deformations in complex geometries. However, this potential does not seem to have been completely exploited in the present work. Although not explicitly stated, the authors seem to be using a linear viscoelastic constitutive relation for the tissue material. Tissue undergoing large deformations during morphogenesis may not however remain in this linear regime. Can the authors consider hyperelastic constitutive relations in their model and does this show better agreement with experiments? In*

*particular, it has been shown that epithelial cells are “superelastic” under osmotic stress (joint work by Arroyo and Trepat labs), that is they can stretch several times their original volume.*

We thank the reviewer for the interest given to our work and we agree with the remark of the reviewer concerning large deformation during morphogenesis.

In the specific case of joint work by Arroyo and Trepat labs (Nat Commun. 2023 Jul 7;14(1):4014. doi: 10.1038/s41467-023-38879-7), they worked with large deformation but with a completely different experimental model, curved epithelia with controlled geometry. Indeed, the establishment of the tissue is driven by the substrate, which is not the case in our experimental model. In their study, they used the MDCK epithelial cell line cultivated on 2D photopatterned PDMS substrates displaying a 3kPa Young’s Modulus while, the stiffness of the Corning Matrigel matrix we used for the culture of the 3D human colon organoids is approximately of 0.05 kPa (Lambricht, L. et al. The type and composition of alginate and hyaluronic-based hydrogels influence the viability of stem cells of the apical papilla. Dental Materials, 30(12):e349-61 (2014)). Indeed, the establishment of the

tissue in the work by Arroyo and Trepat is driven by the substrate, which is not the case in our experimental model.

In our observations, the level of deformation and the change in the volume during the human colon organoid morphogenesis (Figure 1) do not require the large deformation hypothesis. We are indeed modeling deformation in a quasistatic simulation.

It would possibly be feasible to increase the cell behavior law by incorporating additional physical phenomena, such as superelasticity or porosity, as suggested by R1. As illustrated in the following figure and its related table, these laws can be integrated into our model. However, a challenge remains: determining the value of the constitutive parameters. Indeed, as the nonlinearity and the number of parameters in a behavior law increase, errors in estimating these parameters can have a more pronounced impact on the accuracy of the calculations. Therefore, to ensure the model's relevance to its intended application, it is crucial to obtain precise estimates of its parameters. In our case, this means knowing the bulk modulus and void ratio for porosity, or the transformation strain for superelasticity. However, experimentally determining these parameters on a 3D human colon organoid model is highly complex and can lead to significant uncertainty in the results*.*

However, as an example to answer R1 question, the following figure and table demonstrate the potential of our approach by incorporating superelasticity (and porosity) into our model, using parameters adapted from the Abaqus documentation. In practice, we determined the parameters through trial and error to ensure that the numerical convergence properties remain consistent with those of the elastic model. As we can see, this does not lead to an improvement of the model. *Thus, in the manuscript, we decided not to increase more parameters for the registration in our model and did not include this result. However, we now have discussed this possibility for future inference and registration usages of our model. From lines 418 to 421 in the discussion section, you can now read (Additions in the text are underlined):*

*‘The potential of our approach is important since FEM allows the behavioral law of our model to be honed, without the limitation of computational complexity. It could be however possible to add more complexity using other material properties already present in the Abaqus solver like hyper elasticity or porous elasticity as for example Latorre et al have shown that cells can be superelatic under osmotic stress^62’^.*

Moreover, we are aware that potential future evolution of the model are possible, such as adding FEM capability to address cell/cell contact, cell specification (with specific material properties to a set of cells) or complexification of the behavior law (by adding more material properties). *We have thus further discussed this point in the discussion as they can represent the perspectives of this work. From lines 422 to 428 in the discussion section, you can now read (Additions in the text are underlined):*

‘Another idea is to optimize the problem with individual mechanical properties such as viscoelasticity for each subcellular component of each cell (cytoplasm, membrane, cytoskeleton; Figure 2) in order to infer these local properties based simply on cellular shapes inside the organoid. The idea would be to study each cell individually, which would make it possible to address more specifically the impact of mechanosensor signals on each cell, whether intrinsic or extrinsic to the cell. Indeed a reverse approach, from deformation observation to stress and strain evaluation, could help to evaluate the evolution of material properties at cellular level.’

**Figure: Test of the porous elastic and the super elastic states:** Human intestinal organoid FEM model simulation of deformations occurring from immature morphology to mature morphology during polarization event. A/ Schematization of the finite element analysis loading sequence used in the in silico model. Osmotic pressure is applied to different cellular surfaces of the in silico organoid model (*See also supplementary table 1 for the applied parameters)*. B/ Results of the FEM simulation on a 390 cell virtual organoid for step 1 (a, d), step 2 (b, e) and step 3 (c, f), equivalent respectively to the immature, intermediate, and mature organoid morphology. Stress (MISES, a,b,c) and Strain deformation (d,e,f) plots at subcellular resolution at the global view (basal) and the cut view (apical) of the in silico organoid model. C/ Organoids morphology descriptors resumed with four different distribution box plots for the cellular volume, apical surface area, apical/lateral aspect ratio, and spreading distance distribution for one in silico organoid at step 1, step 2, and step 3. Box and whiskers represent the quartiles.

**Table: Parameters used to test the porous elastic and super elastic states.**

- ***2:*** *During osmotic expansion, or active contraction, cells regulate their volume through fluid flow. This should lead to poroelastic effects in their dynamics. Can the authors implement this in their FEM model?*

As suggested by R1 porous-elastic law of behavior governed by the osmotic load could be used to model poroelastic effects. As seen in the above figure, we integrated this law with the addition of porous elasticity parameters to the volumic elements constituting the cytoplasm of our virtual cells, as listed in the joined table. As said above, *these parameters did not lead to a better fit of either the individual cell morphology (New figure D) or the global organoid morphology of the synthetic organoid model compared to those of the biological model. Thus, we decided not to include them in our modeling approach in order to minimize the potential errors. However, as seen above, we mentioned in the discussion that poroelastic parameters could be used in the model.*

- ***3:*** *How general and biologically significant are the kind of shape transitions demonstrated for this system? Does the immature to mature structural changes shown in Fig. 1C have some biological function, and is it generic in many kinds of epithelial tissue? For example, It is known that in the Drosophila oocyte (see e.g. Brigaud et al. 2015 Biology Open), such squamous and columnar cell shapes can co-exist. Can the authors show such a possible co-existence in their model, as was indeed predicted in Ref. 32 that the authors cite? Based on the model of Ref. 32 (Hannezo et al) and experimental studies such as by Widman and Dahmann, Journal of Cell Science 2009, it was theoretically proposed by*

*Dasbiswas, Hannezo and Gov, Biophysical J 2018, that chemical signaling gradients (“morphogens”) can influence cell mechanics and shape. A discussion of such mechano-chemical effects and how to incorporate them in the FEM framework can strengthen the manuscript. Without a discussion of the biological relevance of the types of deformation described here, and the general applicability of this model to systems beyond the one case it has been tested on, the paper does not seem very complete.*

Regarding the structural changes observed in the intestinal epithelium between the immature and the mature states, as shown in the supplementary figure provided (Supplementary figure 1), they correspond to the acquisition of mature/final functions of the monolayer epithelial tissue. During the transition from the "immature" stage, where the epithelium is not yet polarized and does not display differentiated/specialized intestinal cells, to the "mature" stage, the cells polarize and differentiate to acquire their specific final functions, as illustrated in this supplementary figure with the visualization of the enterocytes thanks to their specific

cytokeratin 20 staining (ck20-red staining). We named 'intermediate' the organoid morphologies corresponding to an intermediate state between these two morphologies/states. The immature state observed in culture corresponds to a fetal-like associated phenotype of the organoids as exemplified in this figure from the Graphical abstract from Sprangers, J., Zaalberg, I.C. & Maurice, M.M. Organoid-based modeling of intestinal development, regeneration, and

repair. *Cell Death Differ* **28**, 95–107 (2021). [https://doi.org/10.1038/s41418-020-00665-z.](https://doi.org/10.1038/s41418-020-00665-z)

Indeed, acquisition of polarity and its linked modulation of the cell functions in a commun process in epitheliums (Campanale JP, Sun TY, Montell DJ. Development and dynamics of cell polarity at a glance. J Cell Sci. 2017 Apr 1;130(7):1201-1207. doi: 10.1242/jcs.188599). Regarding organoids, these morphological changes have been reported for different types of tissues such as pancreas and

cholangiocarcinoma for example (Hof, L., Moreth, T., Koch, M. et al. Long-term live imaging and multiscale analysis identify heterogeneity and core principles of epithelial organoid morphogenesis. BMC Biol 19, 37 (2021). doi.org/10.1186/s12915-021-00958-w). In consequence our in silico model could certainly be used to better understand the mechanical properties of other monolayer epithelium organoids (meaning organoids from the entire digestive tract, but also uterus, ovary…) but also other biological models.

Regarding the specific modeling of different cell types, such as squamous and columnar cells, the limitations due to the experimental conditions (staining to visualize in the meantime nuclei, actin or membrane by live microscopy on the human colon organoids) to follow the organoid morphology transition does not allow us for the moment to specifically observe the different cell type. The work done by Hannezo and colleagues is mainly done on murine model using tissues from transgenic mice expressing cell type specific fluorescent reporters.

We do agree with the fact that chemical gradients (morphogens) are involved in the regulation of cell shape and that it would be interesting to be able to study and model their effects on the Human colon epithelium. However, the human colon organoid model cultivated in Matrigel is not the best- suited model since under its experimental condition the environmental chemical conditions are similar all around the organoid, not generating any specific gradient. It would be interesting to perform a similar study on human colon organoid culture established in a gut-on-chip system reproducing the mechanical and chemical environmental conditions of the human tissue *in vivo.*

*As suggested by R1, we incorporated these different points in the discussion of the manuscript (from line 432 to line 443): ‘It would be interesting to challenge this FEM approach to model cell specification during morphogenesis, for example in the vertebrate neural tube or drosophila oocyte where gradient of morphogen and specification are well described^63^ but the involvement of individual cell mechanics rest to determine. Another appropriate biological spherical model is the ascidie that combines the advantage of poor cell number, nice imaging facilities and a reach description of cell lineage acquisition ^64,65^. However, regarding more specifically the human colon, to study how chemical gradients (morphogens) are involved in the regulation of cell shape, the human colon organoid model*

*cultivated in Matrigel is not the best-suited model since under its experimental set-up the environmental chemical conditions are similar all around the organoid, not generating any specific gradient. Thus, in the future, it would be interesting to perform a similar study on human colon organoid culture established in a gut-on-chip system reproducing the mechanical and chemical environmental conditions of the human tissue in vivo and where the cell compartmentalization of the different cell*

*types as well as the chemical gradients can be reproduced’.*

- ***4:*** *Most experiments described seem to be on the wild type of the same epithelial tissue. Can the authors do knockdown or drug treatment experiments that will allow a more complete exploration of the parameter space in their model?*

It would possible to edit genes in human organoid culture, but the only efficient way is to use lentiviral transfer that is intrusive and could in result affect the mechanical properties of the cells, chiefly in the case of the study of very young organoid cultures. Here we preferred, as mentioned by the reviewer, to use Forskolin, a drug that mimics the osmotic stress (Figure 6) and that is frequently used for mechanical studies on epithelial tissues.

- ***5:*** *In figure 4Ce, it seems strange that active contraction does not reduce the apical area relative to the initial state. Can the authors intuitively explain this observation?*

We do understand the non-intuitive observation that led to the construction of this cell. In this specific case where we do not move other load parameters, such as the pressure imposed on the apical surface, the contraction of a small region leads to invagination. Additionally, the elasticity of the lateral membrane is high, leading to an increase in the apical surface area of surrounding cells. Figure 4 can be seen as an example of what happens when we perturb individual load types. A future challenge will be to adapt each load regionally to best accommodate the deformation. Here the global deformation that we impose on the overall morphology of the organoids is not really adapted to the study of the specific deformation of a single cell.

#### Minor comments

- *1’: The language of the manuscript needs improvement. There are several unclear sentences or unconventional usages in English that may make understanding difficult. For example, the use of*

*“increment” in “incrementing a finite element model” in lines 69-70 is not familiar to me. Sentence in 364 is grammatically incorrect (wrong word order). There are typos in lines 76, 305 and several other places too long to list. The authors should carefully proofread their manuscript at the revision stage.*

A professional translator has proofread and edited the manuscript.

- *2’: It should be made clear at the outset that apical/basal surfaces correspond to the inner/outer surfaces in this geometry. This may not be clear to non-specialists not familiar with the specific model system used here. I am seeing this mentioned first in line 286 of the manuscript, which is a bit too late.*

We do agree that a misunderstanding can appear for non-specialists not familiar with the specific model system we used. To clarify the orientation of the cell and the correspondence of apical/basal surfaces, in the figures and in the text (starting from line 106 when we first describe the model) we have now specified apical-luminal side and basal-matrix side. We cannot use the term ‘inside’ or ‘outside’ as for specialists the apical side corresponds to the outer world and the basal side to the inner body.

In detail, clarifications have been added:

- *from line 106 to 112*, ‘They evolve from an ‘immature’ spherical morphology (Fig. 1B, T0) with a flat epithelium (flattened nuclei, elongated and flattened cells) and a central lumen (L) to a ‘mature’ morphology (Fig. 1B, T0 + 180 minutes) displaying a thicker polarized epithelium (reduced apical (facing the lumen) and basal (facing the matrix) cell pole areas, associated with elongation of the lateral sides and swelling of the apical membrane, nuclei in basal position (near the basal pole of the cell), a reduced central lumen (L), and deformations resulting in a less spherical global organoid morphology’
- *from line 240 to 242:* ‘Indeed, forces at the top surface of the cell (apical-luminal side) are linked to the need for apical contraction during epithelial invagination, while forces at the bottom of the cell (basal-matrix side) are more commonly associated with cellular expansion in response to a disassembly of the basal actin cytoskeleton^46’^.
- *3’: Maybe mark the parameters in table 1, diameter and thickness ratio, in Fig. 1 to help the reader.*

This has been done:

**Response to Reviewer #2:**

- **1 :** The paper is poorly composed, so that it is difficult to determine exactly what the method is. The introduction gives a lot of motivation, but no roadmap for what is done in this study.

Reviewer 2 mainly highlights issues regarding the form of our manuscript. As we are not native English speakers, we do agree that our writing in English has to be improved. The manuscript has been corrected and edited by a professional translator.

- **2:** Tables of parameters are presented without justification, and in some cases are presented without appropriate units.

We gave more justification or explanation regarding the choice and use of the parameters.

Regarding Table 1 **(Parameters extracted from live observation and analysis of the biological human colon organoid model)**, from line 103 to 106, we added: *‘Table 1 provides the geometrical parameters characterizing the biological organoid model such as their diameter, the estimated number of cells forming the organoid based on the live microscopy observation, the organoid thickness that reflects its maturation stage but also the number of cells that have been used to collect their individual geometrical properties’.*

Concerning Table 2, which title is ‘**Rheology model parameters: definition of the parameters used for the organoid architecture equilibrium resolution using Tyssue quasi-static solver (AVM simulation)**’, we did not make any change since, from line 176 to 181, it was already explained that *‘We solved the system by adapting mechanical parameters (Table 2), determined to match the observed biological morphologies of the organoids as closely as possible (Fig. 1). This led to deformation of the vertex organoids (from Fig. 2Ca, d, c to Fig. 2Cb, e, and h) via vertex displacement. We were then able to obtain information on individual cells concerning their volume, their apical and basal areas, their lateral areas, and the aspect ratio due to the evolution of organoid morphology (Fig. 2C c, f, and i).*

Regarding Table 3 entitled ‘**Material parameters used in the *in silico* organoid FEM model obtained by manual optimization based on both relevant global organoid morphology simulations and the literature’, we did not add more explanation as from line XX et XX we explained that ‘**Using the FEA solver Abaqus, we decomposed the shape changes by implementing three types of stresses/loads

to obtain elementary morphological deformations in the *in silico* model, generated on the basis of material properties (Table 3)’ however, we changed the units to the international system units.

- **3:** It is not clear whether there have been any tests of model sensitivity. Some parameters are given in a.u. without explanation.

The remark concerning the tests of model sensitivity is surprising. Indeed, there are two ways of interpreting the notion of sensitivity highlighted by the reviewer.

The first concerns the sensitivity of the FE mesh. In a study using a finite element model, it is important to obtain a model that is independent of the mesh. In other words, it is preferable to avoid a model whose results (deformations, stresses, and displacements) depend directly on the size of the elements used. To achieve this, it is necessary to carry out a mesh sensitivity study: run the same analysis several times, refining the mesh with each analysis (i.e. reducing the mesh size). However, it is impossible to apply such a procedure in our case since the mesh size is directly given by the in vivo measurements. This is precisely the contribution of our approach.

The second concerns the global sensitivity analysis of the model parameters themselves (line and surface tension, contractility, area and volume elasticity). The aim of such an analysis is to assess the importance of these parameters on the output variables (cell stress and cell strain). We are surprised by reviewer 2’s remark, as such analyses require a very large number of model calls and are therefore inaccessible for EF models. For example, if we consider the very popular Sobol's method [Saltelli, et al. Sensitivity Analysis, https://books.google.fr/books?id=gOcePwAACAAJ, Wiley (2020).

However, for a model with n inputs, each with a Monte Carlo sample of size N, estimating the first- order and total indices requires Nx(2n+1) calls to the model. Based on these considerations, for a model with less than 10 parameters, at least 10,000 samples are needed to ensure convergence of the Sobol index. In our case, this would lead to 10000x(2x5+1)= 110,000 calls to the model, which is totally unfeasible given the computation time involved.

- **4:** Much of the writing is geometrically sloppy, such as (line 66) "each cell is represented by polygons", which leaves the reader wondering what dimension is being referred to, and the confusing "all cells are attached to the basal and apical planes" (line 130).

Regarding the representation of the epithelial cells by polygons it is an usual way to represent them in modeling approach as done in the article ‘Shape transformations of Epithelial Shells’ by Mahin Misra et al., Biophysical Journal, Volume 110, Issue 7, 2016, Pages 1670-1678, ISSN 0006-3495, https://doi.org/10.1016/j.bpj.2016.03.009. Moreover, the design of the polygon and its correspondence to the cell, as well as its components (cytoplasm, membrane, and cytoskeleton) or its orientation is described in the figure 2A. However, to clarify in the text of the manuscript the orientation of the cell(s) and the correspondence of apical/basal surfaces, as said above in response 2’, starting from line 106 when we first describe the model, we have now specified apical-luminal side and basal-matrix side. Changes have also been done on the figures 1, 2 and 4. Finally, the same color code is used all along the figures in the schematic representations of the organoids or in tables, where the apical-lumen side is blue and the basal-matrix side is red.

- **5:** There are also nonstandard terms, like "external solicitation", which do not contribute to the reader's understanding.

The term ‘external solicitation’ has been previously used in other studies such as reference ***15.*** *Kinoshita, N. et al. Mechanical Stress Regulates Epithelial Tissue Integrity and Stiffness through the FGFR/Erk2 Signaling Pathway during Embryogenesis. Cell Reports 30, 3875-3888.e3 (2020).*

In another work that we cited, the term ‘external solicitation’ is used in the title of the article reporting a study using Finite Element Analysis. We cited this study as ***16.*** *Burlacu, L., Dănilă, C., Birnoveanu, T.I., Cimpoeșu, N. & Bujoreanu, L.G. Analyze of shape memory wires behavior under* ***external solicitation*** *using finite elements analysis (FEA). IOP Conf. Ser.: Mater. Sci. Eng. 1037 012031 (2021),*

- **6:** The awkward phrasing and grammatical errors make me wonder if the MS was first written in French, and machine-translated without intervention from a native English speaker. On the other hand, there are awkwardly complex sentences such as "Deciphering the individual contributions of these intertwined cell events into the intestinal tissue architecture establishment necessitates producing a powerful computational model gathering biology, physics, and geometry" (line 58) which suggests AI writing. If the paper is resubmitted, I strongly urge the authors to use a human copy editor.

We did not used machine-translation or AI writing. As said above, now the manuscript has been corrected and edited by a professional translator.

- **7:** The figures need revision. Fonts are too small in many places, and inconsistent color scales are used for comparisons.

Figures have been revised in this way. Fonts have been increased. The color scales correspond to the range of the experimental set-up, as evaluated by the Abaqus software, it would be scientifically incorrect to modify them.

- **8:** Overall, this paper presents a modeling methodology that has some potential, though there are recent papers which do much the same thing but with more accuracy and versatility (such as time dependence).

We do consider time dependence in our work, as pointed out by Reviewer 1 at the very beginning of his comments, and take into consideration the viscoelastic parameters of the tissue. To our knowledge based on the literature, we are not aware of recent papers doing ‘the same thing but with more accuracy and versatility’. As reviewer 2 does not cite any of these articles, it is difficult for us to defend our work in the face of these possible articles.
