# Supplementary material for: Deciphering the interplay between biology and physics with a finite element method-implemented vertex organoid model: A tool for the mechanical analysis of cell behavior on a spherical organoid shell
Source: PLoS Comput Biol. 2025 Jan 10;21(1):e1012681. doi: 10.1371/journal.pcbi.1012681 (PMC11771887; doi:10.1371/journal.pcbi.1012681)
Supplement: Attachment — Submitted filename: review_PlosCompBio_Sep24.docx [file pcbi.1012681.s003.docx]

I appreciate the effort the authors have put in responding to my previous questions, and in carrying out new simulations using poroelastic constitutive relations. However, on re-reading the manuscript, I have some remaining questions and concerns.

1. The main interesting experimental observation that the authors want to address seem to be the cell shape changes shown in Fig. 1. These are nicely quantified in terms of the four metrics in Fig. 1F. But it does not seem like the simulations reproduce these trends in Fig. 4C. For example, cell volume and apical cell area are observed to decrease monotonically with maturation, but in Fig. 4C these quantities go down and up again. So can the authors claim that the FEM model is validated by the experiment?
2. Even if the trends were to be quantitatively captured, there is no physical explanation of why these cell shape changes occur that I could find. The choices for the values of osmotic pressure and contractility in Steps 1-3 are not very well explained. It leaves the reader with the impression that these values were chosen “by hand” to reproduce the experimental sequence of maturation. For example, it is intuitively obvious that switching off lateral contractility from step 1 to 2 should allow cells to increase lateral area and reduce apical:lateral cell aspect ratio.
3. Since the parameter choices do not seem unique, perhaps it is also possible to tune them in the poroelastic model to get a better match with the experimental trends?
4. While I appreciate the efforts of the authors to improve the language of the paper, the new paragraphs they added in the Discussion (underlined) have many grammatical errors, and unclear sentences. They should also have these professionally translated if possible.
