# Supplementary material for: Deciphering the interplay between biology and physics with a finite element method-implemented vertex organoid model: A tool for the mechanical analysis of cell behavior on a spherical organoid shell
Source: PLoS Comput Biol. 2025 Jan 10;21(1):e1012681. doi: 10.1371/journal.pcbi.1012681 (PMC11771887; doi:10.1371/journal.pcbi.1012681)
Supplement: Attachment — Submitted filename: Second Responses Reviewers PCOMPBIOL-D-24-00672-R1.docx [file pcbi.1012681.s004.docx]

**Point-by-point responses to the reviewers’ comments and description of the changes made on the manuscript PCOMPBIOL-D-24-00672-R1 by Laussu et al.**

We acknowledge that the first reviewer (R1) *‘appreciate(s) the effort (we) have put in responding to (his) previous questions, and in carrying out new simulations using poroelastic constitutive* *relations*’. He raised a few new concerns that we have all addressed. We also appreciate that Reviewer 3 recognizes that we developed ‘*a valuable tool for the community’,* and that *‘in general, (he) concurs with the suggested* *interpretations*’. We have considered all the different points he raised and made changes/additions to the manuscript based on them.

We do believe that our responses to the new points raised and the changes made in the revised manuscript will answer all their criticisms. We have edited the text to take account of all the referees' comments and suggestions. In the revised version of the manuscript, we have also modified the text to clarify the referees' concerns.

Regarding English, the manuscript has been proofread and edited by a professional translator.

Regarding the access to our data, we added in the manuscript that all the data have been made accessible. They can be found at <https://github.com/OrganoFEM/organofem_published>, including, in the README a link to four heavy files that cannot be loaded on GitHub. These files are available here: <https://figshare.com/articles/dataset/organofem_dataset_to_test/27275985>.

We also added in the manuscript (new text in blue):

**Code and Raw data availability:** The software, standard parameter files, and tutorials can be found in the GitHub repository: https://github.com/organofem. The "organofem-published" repository is dedicated to this paper and includes a complete and standalone package (full version with Python and C codes). It contains all the necessary codes, along with a guide for installing the required dependencies, with examples such as standard parameter files and a tutorial for testing the software. Documentation is also provided in the form of a README.md file to guide users. The raw data corresponding to this article can be found in the "test/article_rawdata" folder within the GitHub repository.

We do hope that our manuscript will finally reach publication standards in PLOS Computational Biology.

**Specific responses to the Reviewers**

*(The line numbers indicated correspond to the ones on the ‘tracking mode’ version of the manuscript with all the changes visible)*

**Reviewer #1 (R1):** *I appreciate the effort the authors have put in responding to my previous questions, and in carrying out new simulations using poroelastic constitutive relations. However, on re-reading the manuscript, I have some remaining questions and concerns.*

***Responses to R1:***

1. *The main interesting experimental observation that the authors want to address seems to be the cell shape changes shown in Fig. 1. These are nicely quantified in terms of the four metrics in Fig. 1F. But it does not seem like the simulations reproduce these trends in Fig. 4C. For example, cell volume and apical cell area are observed to decrease monotonically with maturation, but in Fig. 4C these quantities go down and up again. So, can the authors claim that the FEM model is validated by the experiment?*

Figure 4C only presents the different types of loads that we sought to model to mimic the biological phenomena generating the deformations observable on the organoids. Therefore, we think that here R1 means figure 5 in which we sought to model, using FEM, a global deformation of an organoid reproducing the global morphological alterations that we can observe on a biological organoid, without initially seeking to fall back on the same range of deformations at the cellular level.

Regarding Figure 5, R1's observations at the cellular level are accurate. The FEM model does not reproduce the biologically observed behavior (the cellular volume and apical surface do not decrease monotonically). However, R1's remark makes us think that at this stage, the description of our approach is not clear, as these criteria are not meant to experimentally validate the behavior of the FEM model but rather to generate a FEM model of a consistent organoid. The validation will be addressed in the experiment with the swelling essay (Figure 6). Indeed, in Figure 5, what we sought to reproduce in the FEM model were the following biological phenomena: a global compaction of the organoid and an increase in the thickness of the epithelium. This results in cellular-level geometric behaviors within the FEM model, where the time evolution does not reflect cellular reality in the biological model. Specifically, inducing a global contraction of the apical cortex while increasing the overall thickness of the epithelium led to an increase in the apical surface due to swelling of the apical surface (Step 3).

In order to better explain our approach, we have modified the text of the paragraph concerning this stage of our study. From line 366 to line 380, you will now read (modifications in blue):

‘As earlier, we then obtained information on individual cells concerning volumes, apical and basal areas, lateral areas, and aspect ratio due to the evolution of the organoid morphology, which appear to be in the same range of values as those observed in the biological model (Fig. 5C). By comparing the results extracted here with those of the biological model, we found that applying FEM on our vertex model allows us to validate the choice of material properties describing the subcellular components. Thus, from this approach, we validated the mechanical behavior laws describing global viscoelasticity and volume expansion in our in silico model. However, while we observed that we cannot reproduce perfectly some cellular morphological properties evolution as for example the cellular volume and apical surface do not decrease monotonically, our approach helped us to identify the range and types of loads (external solicitation^15,16^, active contraction^17^, and osmotic pressure^14^) involved in establishing the overall morphologies of the organoids. We hypothesized that the side effects could be due to the fact that we applied a combination of several loads. Thus, to validate our approach, we decided to test only one load, the osmotic pressure, as we can easily modulate this parameter in the biological model thanks to Forskolin’.

2. *Even if the trends were to be quantitatively captured, there is no physical explanation of why these cell shape changes occur that I could find. The choices for the values of osmotic pressure and contractility in Steps 1-3 are not very well explained. It leaves the reader with the impression that these values were chosen “by hand” to reproduce the experimental sequence of maturation. For example, it is intuitively obvious that switching off lateral contractility from step 1 to 2 should allow cells to increase lateral area and reduce apical:lateral cell aspect ratio.*

As noted by R1, the values for the different loads—such as consecutive osmotic pressure and active contraction—used to model the morphological evolution of the organoid were determined manually. These values were selected 'by hand' to replicate the experimental sequence of maturation. We can provide further explanation of the method used to identify these values:

First, we explored the minimum and maximum values that allowed for a solution of the system in terms of computation, ensuring that Abaqus could find a convergent solution. The next step involved exploring combinations of these loading values to best represent the morphology observed in the initial stage. After validating the first step of the simulation, which involved the inflation of the organoid, we proceeded with the second step of deflation, and finally, the last step, which focused on the enlargement of the organoid shell. This solution is not unique, but it is the best we can achieve in terms of biological relevance for the overall morphology of the organoid.

The second argument of R1 that it is *‘obvious that switching off lateral contractility from step 1 to step 2 should allow cells to increase lateral area'* is valid. We applied the loads intuitively to simulate the process, based on our understanding of the basic mechanisms driving the biological events. Similarly, regarding the osmotic changes, we hypothesized that the increase in cellular volume observed in Figure 1 was the result of increased osmotic pressure inside the cells.

3. *Since the parameter choices do not seem unique, perhaps it is also possible to tune them in the poroelastic model to get a better match with the experimental trends?*

It is true that, to obtain the best possible set of parameters, this problem should ideally be formulated as an inverse problem and solved using numerical optimization algorithms. To set up this inverse problem, we would need time-series data that enables displacement realignment and correlation. However, the non-linearity resulting from the combination of porous elasticity, viscoelasticity, and super-elasticity—tested as suggested by Reviewer 1—makes this calculation too complex.

Tests of arbitrarily chosen combinations of parameters also lead to a majority of non-convergence of the calculation. The parameter definition domain is highly irregular, so it was not possible to perform a recalculation of parameters by the conventional optimization method. For illustration, the complete experiment plan with the 4 parameters at 2 levels indicated in the table below was conducted. Only 6 combinations allowed the convergence of calculation and therefore the achievement of a usable result. We therefore chose to make a manual optimization of the parameters, guided by the first results of this experiment and about ten other complementary trials. The values chosen for the article are derived from a manual optimization in the vicinity of the best set of parameters obtained initially.

Below is the range of values we used in the preliminary design of experiment, and the resulting set of parameters applied to our model:

| **Values** | **Bulk modulus** | **Poisson’s ratio** | **Tensile  limit** | **Void ratio** |
| --- | --- | --- | --- | --- |
| **Min** | 0.005 | 0.27 | 0.012 | 0.55 |
| **Max** | 0.02 | 0.35 | 0.017 | 0.65 |
| **Calculated** | 0.0209 | 0.3299 | 0.0145 | 0.6000 |

We optimized the model as best as possible using the calculated mechanical parameters, but we did not find another solution to the porous elastic problem that produced better results in terms of the global morphology of the organoid.

4. *While I appreciate the efforts of the authors to improve the language of the paper, the new paragraphs they added in the Discussion (underlined) have many grammatical errors, and unclear sentences. They should also have these professionally translated if possible.*

Regarding English, we have taken this point into consideration, the manuscript has been proofread and edited by a professional translator.

***Reviewer #3 (R3)****: The authors suggest a tool for mechanical analysis of the behavior of human colon epithelial cells on an organoid spherical shell. The tool integrates several published or commercial computational tools, including a classic vertex model, previously defined finite elements models in Abaqus, and other tools for image analysis and 3D multicellular displays.*

*I think this could be a valuable tool for the community, and in general, I concur with the suggested interpretations. However, there is a big problem with the way the tool is presented and motivated. The authors also fail to cite prior relevant publications, or poorly refer to them. Furthermore, I find the language and wording used in many parts throughout the MS to be unclear or awkward.*
*Another cardinal issue here is the lack of effort to make it clear that the suggested tool is an integration of many available tools (some commercially). It is written in the methods section and in some parts of the text, yet, it is not spelled out immediately from the beginning. If it will be so, I think, it will make this work more attractive and useful for others. Lastly, and regarding usefulness, the fact that the suggested tool is mainly an integration of many other available tools calls for full availability in the Supp Material or some other cloud service. This should include a detailed installation and instruction guide and an available example data set to run one of the analyses presented in the MS.*

***Responses to R3:***

***General comments:***

- Regarding the ‘*problem with the way the tool is presented and motivated’.*

We agree that indicating as early as possible that our approach uses several freely available tools or a commercially available software could be a good argument for our approach to be of interest to others. Moreover, we agree that giving a quick overview of our experimental approach could help the reader. Thus, as soon as in the abstract, we added this information. The new version of the abstract is (new text in blue).

‘Understanding the interplay between biology and mechanics in tissue architecture is challenging, especially when it comes to 3D tissue organization. Addressing this challenge requires both a biological model that allows observations at multiple levels from cell to tissue, and theoretical and computational approaches enabling the generation of a synthetic model that is relevant to the biological model and provides access to the mechanical stresses experienced by the tissue.

Using the monolayer organoid of human colon epithelium as a biological model, freely available tools (Fiji, Cellpose, Napari, Morphonet or Tyssue library), and commercially available Abaqus FEM solver, we combined vertex and FEM approaches to generate a comprehensive elastic finite element model of the human colon organoid and demonstrated its flexibility. Indeed, we imaged the development of the human colon organoids for 120 hours, which allowed us to follow the evolution of the organoids from an immature to a mature morphology. Based on the extracted architectural/geometric parameters of human colon organoids at different stages of the tissue architecture establishment, we generated organoid active vertex models. However, this approach failed to take into consideration the mechanical aspects involved in the morphological evolution of the organoids. To do so, a finite element method considering mechanical loads mimicking osmotic pressure, external solicitation or active contraction were applied into the vertex model using Abaqus FEM solver. The integration of Finite Element Analysis (FEA) to the vertex model showed a better fit with the biological. Indeed, the FEM model provides a basis for depicting cell shape, tissue deformation, and strain at cellular level due to imposed stresses.

In conclusion, we demonstrated that the combination of vertex and FEM approaches, combining geometrical and mechanical parameters, improves modeling of the alteration of organoid morphology over time and provides a better assessment of the mechanical cues involved in establishing the architecture of the human colon epithelium’.

Moreover, in the introduction of the manuscript (lines 99 to 102), we added earlier references to the Licensed software Abaqus: ‘Among the commonly used software solutions in the industry, Abaqus is an effective option that offers a large number of predefined elements for studying complex physical phenomena. It provides powerful flexibility and modularity to approximate complete partition by polynomial functions on each element.

A little bit later in the introduction, we also add more description of our development (line 113 to 117): ‘A large part of our study aimed to integrate the interface between the AVM and the FEM model by developing python algorithms to translate vertex architecture into elements preexisting in Abaqus. The second part of our algorithmic development aims to extract morphological information from the FEM model by recreating surface mesh allowing the measurement of the cellular surface areas’.

- Regarding the *‘fact that the suggested tool is mainly an integration of many other available tools calls full availability in the Supp Material or some other cloud service. This should include a detailed installation and instruction guide and an available example data set to run one of the analyses presented in the MS’.*

We do agree and, as said above, we give now this information in a GitHub repository. That is mentioned in the manuscript under the paragraph:

**Code and Rawdata availability**

The software, standard parameter files, and tutorials can be found in the GitHub repository: <https://github.com/OrganoFEM/organofem_published>. The "organofem_published" repository is dedicated to this paper and includes a complete and standalone package (full version with Python and C codes). It contains all the necessary codes, along with a guide for installing the required dependencies, with examples such as standard parameter files and a tutorial for testing the software. Documentation is also provided in the form of a README.md file to guide users. The rawdata corresponding to this article can be found in the "test/article_rawdata" folder within the GitHub repository.

***Specific comments:***

1. *Title: – promises something very general, yet the MS provides something much more specific. Please modify.*

*As suggested by R3, we now propose to add in the title the text written in blue: ‘Deciphering Biology and Physical Interactions Using a Finite Element Method-Based Vertex Organoid Model: a tool for the mechanical analysis of cell behavior on a spherical organoid shell.’*

2. *Abstract: (in a way similar to the title) I found it too general and lacking concrete observations and conclusions, not from the biophysics aspect nor from the computational aspect.*

We have taken this point into consideration. As said above, we propose a new version of this abstract. The new text is written in blue.

‘Understanding the interplay between biology and mechanics in tissue architecture is challenging, especially when it comes to 3D tissue organization. Addressing this challenge requires both a biological model that allows observations at multiple levels from cell to tissue, and theoretical and computational approaches enabling the generation of a synthetic model that is relevant to the biological model and provides access to the mechanical stresses experienced by the tissue.

Using the monolayer organoid of human colon epithelium as a biological model, freely available tools (Fiji, Cellpose, Napari, Morphonet or Tyssue library), and commercially available Abaqus FEM solver, we combined vertex and FEM approaches to generate a comprehensive elastic finite element model of the human colon organoid and demonstrated its flexibility. Indeed, we imaged the development of the human colon organoids for 120 hours, which allowed us to follow the evolution of the organoids from an immature to a mature morphology. Based on the extracted architectural/geometric parameters of human colon organoids at different stages of the tissue architecture establishment, we generated organoid active vertex models. However, this approach failed to take into consideration the mechanical aspects involved in the morphological evolution of the organoids. To do so, a finite element method considering mechanical loads mimicking osmotic pressure, external solicitation or active contraction were applied into the vertex model using Abaqus FEM solver. The integration of Finite Element Analysis (FEA) to the vertex model showed a better fit with the biological. Indeed, the FEM model provides a basis for depicting cell shape, tissue deformation, and strain at cellular level due to imposed stresses.

In conclusion, we demonstrated that the combination of vertex and FEM approaches, combining geometrical and mechanical parameters, improves modeling of the alteration of organoid morphology over time and provides a better assessment of the mechanical cues involved in establishing the architecture of the human colon epithelium’.

***3. Line 66 (now 70/71):*** *the end of the sentence is unclear. “Furthermore, their in vitro culture facilitates live observation by microscopy, providing relatively high temporal resolution in the architecture establishment follow-up.”*

We propose to change the sentence for: "Furthermore, culturing them in vitro allows for live observation through microscopy, which offers relatively high temporal resolution when monitoring the development of their architecture”.

***4. Line 69 and Line 76 (now 74 to 83)-Vertex models****: It seems that many important papers on cell shape in development and in curved surfaces, specifically with regard to the vertex model and the concept of multicellular jamming, are not mentioned here. To name a few:*

*1. Bi, D., J.H. Lopez, J.M. Schwarz, and M.L. Manning, A density-independent rigidity transition in biological tissues. Nat Phys, 2015. 11(12): p. 1074-1079.*

*2. Bi, D., X. Yang, M.C. Marchetti, and M.L. Manning, Motility-Driven Glass and Jamming Transitions in Biological Tissues. Physical Review X, 2016. 6(2): p. 021011.*

*3. Atia, L., D. Bi, Y. Sharma, J.A. Mitchel, et al., Geometric constraints during epithelial jamming. Nature Physics, 2018.*

*4. Spurlin, J.W., M.J. Siedlik, B.A. Nerger, M.-F. Pang, et al., Mesenchymal proteases and tissue fluidity remodel the extracellular matrix during airway epithelial branching in the embryonic avian lung. Development, 2019. 146(16): p. dev175257.*

*5. Tang, W., A. Das, A.F. Pegoraro, Y.L. Han, et al., Collective curvature sensing and fluidity in three-dimensional multicellular systems. Nature Physics, 2022.*

*The list goes much further, and I would suggest modifying the introduction such that it will at least acknowledge and place in the proper context (of this paper) this body of literature.*

We have taken this point into consideration. In the introduction, line 75 to 79, we add the sentence ‘Many studies on 2D cultures characterize the properties of cell geometry and the fluidic transitions occurring during tissue remodeling (citing references *Bi, D., J.H. Lopez, J.M. Schwarz, and M.L. Manning, A density-independent rigidity transition in biological tissues. Nat Phys, 2015. 11(12): p. 1074-1079, Bi, D., X. Yang, M.C. Marchetti, and M.L. Manning, Motility-Driven Glass and Jamming Transitions in Biological Tissues. Physical Review X, 2016. 6(2): p. 021011*). The deformation of tissues can be assessed in 2D or 2.5D under experimental conditions and highlights the individual contributions of cell density, cellular organization, and the composition of the extracellular matrix (citing references *Tang, W., A. Das, A.F. Pegoraro, Y.L. Han, et al., Collective curvature sensing and fluidity in three-dimensional multicellular systems. Nature Physics, 2022; Atia, L., D. Bi, Y. Sharma, J.A. Mitchel, et al., Geometric constraints during epithelial jamming. Nature Physics, 2018*)’. Thus, all the above references have been cited in the introduction, except (Spurlin et al.), which does not describe any vertex model and which we found too far from our study.

***5. Line 76 (now 84), “volumetric vertex model” :*** *The authors need to better survey similar tools that combined finite element approach and the vertex model such as Smith, A.M., R.E. Baker, D. Kay, and P.K. Maini, Incorporating chemical signalling factors into cell-based models of growing epithelial tissues. Journal of Mathematical Biology, 2012. 65(3) : p. 441-463.*

*And also survey other studies that proposed alternatives to address the vertex model limitations. For example deformable cell models (DCMs)- Runser, S., R. Vetter, and D. Iber, SimuCell3D: three-dimensional simulation of tissue mechanics with cell polarization. Nature Computational Science, 2024. 4(4) : p. 299-309.*

Now, the study by Smith AM et al. has been cited in the introduction at the end of the sentence (reference 27, lines 87 to 89): ‘Among them, 3D vertex models are well adapted to biological systems as they take macroscopic mechanical characteristics into account while offering cell identity representation’, in complement of the reference 26 (ex 22).

Regarding the paper by Runser et al., we added the citation (reference 69) of this study in the discussion and to better introduce this paper, we changed a the previous sentences from ‘The idea would be to study each cell individually, which would make it possible to address more specifically the impact of mechanosensor signals on each cell, whether intrinsic or extrinsic to the cell. Indeed a reverse approach, from deformation observation to stress and strain evaluation, could help to evaluate the evolution of material properties at cellular level’ to (lines 517 to 524): ‘The aim would be to study each cell individually, allowing for a more specific examination of the impact of mechanosensory signals on each cell, whether intrinsic or extrinsic. In fact, a reverse approach - starting from deformation observation and moving toward stress and strain evaluation - could aid in assessing the evolution of material properties at the cellular level within epithelial tissues. In the future, it could be interesting to evolve our model by coupling the vertex model formulation with other methods, such as deformable cell models (DCMs), which have recently been proven to be promising tools for creating virtual organoids^69^.

**6. *Line 163 (now 184):*** *It is not completely clear how the 400 deposited seeds are translated to the 2.5D Voronoi structure. Can the authors describe in an exact and specific fashion what was done? That is - specifics of 3d tessellations, specific transformation, specific codes used.*

As described line 657, the seeds are distributed using a normal distribution function at the surface of a normalized sphere. To give more details in the way we did it, and explain earlier, in the paragraph where we describe the seed deposit, we added this sentence (lines 192 to194): ‘To deposit the seeds, we used a spherical function and the Golden Angle to create a Fibonacci lattice on the surface of the normalized sphere (radius =1). A normal deviation around this function is applied to achieve a non-uniform distribution of points’.

Information can be found in the code ‘create_AVM_organofem.py’ (please go to our Github (https://github.com/organofem) under the function ‘spheric3D()’.

***7. Line 165 (now 196):*** *Do you mean that each cell had the same -yet scaled- polygon describing its 2D apical and basal surfaces? Please better explain.*

We agree with R3. We changed this sentence from: “Finally, we duplicated and connected two copies of the 2.5D shell, creating a closed 3D monolayer spherical tissue.” to ‘Finally, we duplicated and connected two copies of the 2.5D shell through spherical dilation, forming a closed 3D monolayer tissue where the apical and basal faces of each cell share a similar shape’.

***8. Line 175 (now 208)****: “comparable to a behavior law” – not clear.*

A behavioral mechanical law typically refers to a set of principles or equations that describe how materials or structures respond to external forces or stimuli based on their mechanical properties. This term can be used in various fields, such as biomechanics or material science, to define the relationship between forces (like stress, strain, pressure, or deformation) and the resulting behavior of the material, tissue, or structure. A behavioral mechanical law governs the mechanical behavior of a system, determining how it will respond when subjected to mechanical forces, based on inherent properties and conditions.

We changed “comparable to a behavior law” for “the mechanical behavior law governing the system”.

***9. Line 191 (now 222 to 224):*** *It is not clear what “closely as possible” means. Can the authors better describe how these parameters were set, and to what extent are the simulations sensitive to these?*

As explained in the response to R1, the parameters of the rheology model were determined throughout manual optimization to obtain cell deformation approximating the characteristics of the global organoid morphology, notably the radius and thickness of the organoid. This approach ensured that the overall essential dimensions of the organoid were preserved during the modeling process.

As seen in our response to R1, we proposed a description of our manual optimization approach for the study of the impact of porosity in the organoid morphology. Please see our response to the third point raised by R1. To clarify the text, we changes the sentence from ‘We solved the system by adapting mechanical parameters (Table 2), determined to match the observed biological morphologies of the organoids as closely as possible’ to We solved the system by adapting mechanical parameters (Table 2), determined to match as close as possible the observed biological morphologies of the organoids.

***10. Line 195 (now 228):*** *the title of table 2 is “Rheology model parameters…” but it basically contains only (small deformations) elasticity parameters. There are no viscosity or time-rate-dependent parameters.*

We agree again with this point. Indeed, it is a quasistatic solver performing a gradient descent for energy minimization. Thus, we replaced ‘Rheology model’ by **Active Vertex Model’.**

***11. Line 208 (now 241):*** *like in the introduction, it is not clearly described what is the FEM approach used here? What are the differences between older approaches, and this suggested new approach?*

We are not developing a new finite element method (FEM) from a software perspective but rather focusing on innovation in how to design cells and define mechanical parameters, such as contractility, within existing FEM frameworks.

Our approach involves using FEM to better capture the mechanical behaviors of cells, particularly by refining key parameters that influence how cells contract and interact. This allows for more accurate modeling of biological processes like cell deformation, movement, and mechanical responses to forces. By concentrating on cell-specific mechanical features, we aim to improve the precision of simulations in biological contexts, without the need for completely new software solutions. This focuses on redefining how FEM is applied to cell design and mechanics brings new insights into the biomechanical properties of tissues and cellular systems.

We hope that the changes made to the manuscript based on R3’s comments, and as indicated in our response to R3, will better explain our approach.

***12. Line 211 (now 245):*** *It is not clear what constraints are the source of the problem, as these constraints are repeatedly mentioned in the coming lines.*

We introduced the description of the constraints with the exploration of the loads in Figure 4. We now mentioned them in the text: ‘To take these constraints (osmotic pressure^18^, external solicitation^19,20^, and active contraction^21^) into account…’. At this stage we talk about global physical law that cannot be reduced to a simple energy minimization paradigm.

***13. Line 212 (now 246):*** *“VM” - Above it was termed AVM.*

It was previously referred to as the Active Vertex Model (AVM) because we used the vertex model's geometry in Fig. 2, employing a quasi-static solver to minimize the energy of the system. In the AVM, mechanical descriptions are provided by tensors at the vertices (see Table 2). After testing this approach, we proposed integrating the Finite Element Method (FEM) to develop a FEM model that uses the Vertex Model (VM) as its geometric foundation. As a conclusion, here we keep VM because we are talking of the geometry.

***14. Line 218 (now 248):*** *At this point, I believe you mean the AVM model. It needs to be consistent throughout the text.*

As presented in the precedent answer, here we are using geometry (VM) as a based to create the elements.

***15. In Fig 3A*** *please change the title to Active Vertex Model (AVM) - needed to maintain consistency.*

We changed the title, which is now: Finite element method in incrementation on an active vertex model (AVM) of organoid. In this figure we conserved AVM because it is a general vision of the method, and it is possible to couple the two approaches.

***16. Line 222 (now 255):*** *You can also do it using a classic vertex model. You should emphasize that here, you also add the cell interior resistance, and by combining a continuous approach, you get a full stress tensor.*

Our strategy was to utilize the 3D Vertex Model (VM) approach to generate an in silico organoid. To access mechanical properties, we incorporated a finite element method (FEM) into this vertex model. For studying epithelial tissues, the vertex model is widely used as it represents a set of cells through points connected by segments (Fletcher AG, Osterfield M, Baker RE, Shvartsman SY. "Vertex Models of Epithelial Morphogenesis." Biophys J. 2014 Jun 3; 106(11):2291-304). This point-based system serves as the foundation for minimizing an energy function, determining the equilibrium positions of the cell assembly. The 3D positions of the points are decision variables in an optimization problem, which, once solved, provide optimized positions. From these positions, structural and geometrical information, such as cell areas or equilibrium volumes, can be computed.

However, while the vertex model provides valuable geometrical information, it lacks the ability to measure mechanical properties. Another challenge, revealed in the AVM application (Figure 2), arises from the complex elastic response associated with target values of volume, area, or perimeter in AVM (Hernandez A, Staddon MF, Bowick MJ, Marchetti MC, Moshe M. "Anomalous Elasticity of a Cellular Tissue Vertex Model." Phys Rev E. 2022 Jun; 105(6-1):064611).

Our goal is not to invalidate the Vertex Model, which serves as a useful representation in configuration space and a first-step model for our approach, but rather to enhance it. By integrating FEM analysis, we can study various stress components, such as plane stress, shear stress, hydrostatic stress, and anisotropic stress related to deformation.

The potential of our approach is significant, as FEM enables refinement of the model's behavior without being hindered by computational complexity.

***17. Line 223 (now 257):*** *There are also other metrics to translate the local stress tensor to a local scalar, for example, the trace of the tensor (a way to quantify the average tension). What is the incentive to use von Mises stress? For example, in classical engineering, von Mises stress is convenient for formulating safety criteria.*

Different metrics can be employed to reduce a global constraint tensor into a single equivalent constraint value. One widely recognized metric is the Von Mises stress criterion, which aggregates the different stress components acting along various directions into a single scalar value. This scalar value is particularly useful for evaluating the stress levels in materials, including anisotropic ones.

Since the Von Mises criterion is one of the most commonly used methods in classical mechanical engineering, we propose its adoption in the present work to ensure a well-established and widely accepted approach. By introducing this familiar criterion, we aim to facilitate its application in the biology research community, providing a common framework for evaluating stress levels in various biological contexts.

***18. Line 230 (now 256):*** *the first sentence is redundant at this point in the MS: ‘As our model focuses on the use of living material, we characterized the interplay between tissue and cell deformations to design our multi-scale model ranging from the morphology of the individual cell to the global organoid morphology’.*

To be less redundant, we now changed this sentence by: *‘Our model analyzes living material by studying the interaction between tissue and cell deformations. This informs a multi-scale model that encompasses both individual cell shapes and the overall organoid structure’.*

***19. Line 232 (now 263):*** *Not clear. Do you mean an observed non-uniform curvature distribution along the organoid surface?*

Since we can observe three types of local epithelial morphological alterations in the mature organoid, to clarify our remarks, we have modified the text and now propose: ‘*We observed a non-uniform curvature distribution along the organoid shell, which can be classified into three different types of alterations’.*

**20. *Line 233 (now 269):*** *‘The first presents curvature of the tissue with no significant change in cell shape or thickness (Fig 4Aa)’. It is not clear what is “presents curvature”. Also, “no significant change in cell shape or thickness” is not clear. Do you mean no heterogeneity of cell shape or thickness?*

We admit that “presents curvature” is not clear and that talking about “heterogeneity” is better suited to what we want to describe. As a result, we changed the sentence by: ‘*The first scenario displays the curvature of the tissue without any heterogeneity of the cell shape or thickness*’.

***21. Line 244 (now 281):*** *“Solicitation” is a somewhat awkward word here. Do you mean “stimulation”? I would consider revisiting throughout the text and the relevant figures.*

In mechanics, solicitation refers to the application of external forces or loads to a material or structure. This term encompasses the various types of stresses (such as tensile, compressive, shear, and torsional stresses) that a material experiences because of these applied forces.

The term ‘external solicitation’ has been previously used in other studies such as reference **15.** Kinoshita, N. et al. Mechanical Stress Regulates Epithelial Tissue Integrity and Stiffness through the FGFR/Erk2 Signaling Pathway during Embryogenesis. Cell Reports 30, 3875-3888.e3 (2020). In another work that we cited, the term ‘external solicitation’ is used in the title of the article reporting a study using Finite Element Analysis. We cited this study as **16.** Burlacu, L., Dănilă, C., Birnoveanu, T.I., Cimpoeșu, N. & Bujoreanu, L.G. Analyze of shape memory wires behavior under external solicitation using finite elements analysis (FEA). IOP Conf. Ser.: Mater. Sci. Eng. 1037 012031 (2021).

***22. Line 298 (now 335):*** *‘Thus, to facilitate modeling, we defined a homogeneous actin concentration via the expansion coefficient and an inhomogeneous actin activity via a load expressed by a temperature on the in silico model’. The term “temperature” can be confusing to many, and thus another reason to better explain the entire FEM approach here.*

We changed the sentence by: ‘With the same objective of simplification of the modeling process, we defined a homogeneous actin concentration using an expansion coefficient, while inhomogeneous actin activity was represented by a load influenced by temperature in the in silico model’. Following, we added a supplemental sentence to further highlight the uniqueness of our model: “A particular feature of our approach is that cell contractility, specific of a biological system activity, can be finely controlled in the FEM model using a specific load already described in the Abaqus software behavior mechanical laws.”

**Minor comments:**

***In supp fig 2****, in the right green box, change to “cells centers”.*

Thank you for highlighting this error, we corrected ‘Centers cell’ to ‘Cells centers’

***Line 118 (now 138):*** *it should be the organoid shell (or epithelial monolayer) thickness.*

We added ‘shell’ for ‘organoid shell thickness’.

***Line 123 to 128 (now 143 to 149):*** *the sentence ‘They evolve from an ‘immature’ spherical morphology (Fig 1B, T0) with a flat epithelium (flattened nuclei, elongated and flattened cells) and a central lumen (L) to a ‘mature’ morphology (Fig 1B, T0 + 180 minutes) displaying a thicker polarized epithelium (reduced apical (facing the lumen) and basal (facing the matrix) cell pole areas, associated with elongation of the lateral sides and swelling of the apical membrane, nuclei in basal position (near the basal pole of the cell), a reduced central lumen (L), and deformations resulting in a less spherical global organoid morphology (Fig 1B, 1C, 1D)‘ is too long.*

We proposed instead: ‘The organoid evolve from an "immature" spherical morphology (Fig 1B, T0), characterized by a flat epithelium with flattened nuclei and elongated cells, along with a central lumen (L), to a "mature" morphology (Fig 1B, T0 + 180 minutes). In the mature state, the epithelium becomes thicker and polarized, with reduced areas at both the apical (facing the lumen) and basal (facing the matrix) poles. This maturation is also marked by elongation of the lateral sides, swelling of the apical membrane, nuclei positioned near the basal pole, a smaller central lumen, and overall deformations that result in a less spherical organoid structure (Fig 1B, 1C, 1D)’.

***In table 1:*** *please define what is “thickness ratio”.*

The thickness ratio is relative to the radius of the final organoid. Radius of the organoid/(radius basal-radius apical). This had been added to the text, line 159).

***Line 144 (now 172):*** *Like was done in the figure caption- If you choose to specify the tool for 2D segmentation then you should probably also specify the tool for 3D reconstruction.*

As written from line 171, Cellpose 2.0 has been used for both 2D segmentation and 3D reconstruction: ‘B*ased on the organization of the actin-myosin cytoskeleton and its fluorescent staining (Fig 1Ea), we performed 2D segmentation (Fig 1Eb) followed by 3D reconstruction of the organoid using Cellpose 2.0 ^30,31^ (Fig 1Ec)*’. To make it more understandable, we changed the sentence by: ‘Based on the organization of the actin-myosin cytoskeleton and its fluorescent staining (Fig. 1Ea), we used Cellpose 2.0^35,36^ to perform 2D segmentation (Fig. 1Eb) and then for the 3D reconstruction of the organoid (Fig. 1Ec).

***Line 170 (now 203):*** *do you mean” thickness ratio”?*

*Yes, indeed. We replaced ‘thickness’ by ‘thickness ratio’*

***Line 238 (now 275):*** *What is FEA? Not previously defined.*

FEA stands for Finite Element Analysis (FAE). We wrote the full text in the newly added text in the abstract (line 40), and also on line 275 when the Abaqus FEA solver is first mentioned.
