# Supplementary material for: Deciphering the interplay between biology and physics with a finite element method-implemented vertex organoid model: A tool for the mechanical analysis of cell behavior on a spherical organoid shell
Source: PLoS Comput Biol. 2025 Jan 10;21(1):e1012681. doi: 10.1371/journal.pcbi.1012681 (PMC11771887; doi:10.1371/journal.pcbi.1012681)
Supplement: Attachment — Submitted filename: Response to the Editors.docx [file pcbi.1012681.s005.docx]

**Response to the Editors’ last comments:**

As seen in the cover letter and as required, we incorporated our answers to the reviewers (underlined text) in this revised manuscript:

**Incorporation made from the first round of reviewing:**

***From reviewer 1 (R1) questions:***

- Questions 1 and 2 regarding the incorporation of superelasticity or porosity into our model: Our response (italic text above) has been incorporated into the text of the **edited manuscript** from line 368 to line 396. This includes the table presenting the different parameters used to test the porous elastic and superelastic states (Table 4) and the figure presenting the results (please see the ‘new’ figure 6):

*“Moreover, our observations indicated that the level of deformation and the change in volume during human colon organoid morphogenesis (Fig. 1) do not require the large deformation hypothesis. Therefore, we modeled deformation in a quasistatic simulation, using a linear viscoelastic constitutive relation for the tissue material. However, during morphogenesis, tissue may undergo large deformations, thereby implying a nonlinear regime. We consequently tested putative superelastic constitutive relations in our model.*

*Therefore, increasing the cell behavior law might potentially be feasible by incorporating additional physical phenomena, such as superelasticity or porosity (Table 4). As illustrated in Figure 6, these laws can be integrated into our model. However, determining the value of the constitutive parameters remains a challenge. Therefore, as the nonlinearity and the number of parameters in a behavior law increase, errors in estimating these parameters can have more pronounced effects on calculation accuracy. To ensure the model's relevance to its intended application, obtaining precise estimates of its parameters is crucial. In our case, the bulk modulus and void ratio for porosity, or the transformation strain for superelasticity, must be known. However, experimentally determining these parameters on a 3D human colon organoid model is highly complex and can lead to substantial uncertainty in the results.*

*As an example, Figure 6 and Table 4 demonstrate the potential incorporation of superelasticity (and porosity) into our model, using parameters adapted from the Abaqus documentation. In practice, we determined the parameters through trial and error, to ensure that the numerical convergence properties remained consistent with those of the elastic model. As indicated, doing so does not lead to model improvement.*

**Table 4: Parameters used to test the porous elastic and superelastic states**

|  |  | **Elastic** | | | | |
| --- | --- | --- | --- | --- | --- | --- |
| **Material** | **Element type** | **Young modulus (MPa)** | **Poisson ratio** | **Mass density (kton/m^3^)** | **Expansion** | **Specific heat** |
| **Cytoplasm** | **C3D10M** |  |  | 7,80E^-09^ |  |  |
| **Apical membrane** | **STRI65** | 0,013 | 0,33 | 7,80E^-09^ |  |  |
| **Basal membrane** | **STRI66** | 0,25 | 0,38 | 7,80E^-09^ |  |  |
| **Lateral membrane** | **STRI67** | 1,00E^-06^ | 0,38 | 7,80E^-09^ |  |  |
| **Apical cortex** | **T3D3T** | 0,014 | 0,31 | 7,80E^-09^ | 0,03 | 200 |
| **Basal cortex** | **T3D3T** | 0,22 | 0,38 | 7,80E^-09^ | 1,50E^-05^ | 400 |
| **Lateral cortex** | **T3D3T** | 5,00E^-06^ | 0,38 | 7,80E^-09^ | 6,00E^-08^ | 400 |
| **Lumen** | **C3D4** | 4,00E^-06^ |  | 1,00E^-09^ |  |  |
|  |  |  |  |  |  |  |
|  |  | **Porous elastic** | | | |  |
| **Material** | **Element type** | **Log bulk modulus** | **Poisson ratio** | **Tensile limit** | **Void ratio** |  |
| **Cytoplasm** | **C3D10M** | 0,02 | 0,33 | 0,015 | 0,6 |  |
|  |  |  |  |  |  |  |
|  |  | **Superelastic** | | | | |
| **Material** | **Element type** | **Young modulus (MPa)** | **Poisson ratio** | **Transformation strain** | **Start loading** | **End loading** |
| **Basal cortex** | **T3D3T** | 0,014 | 0,31 | 0,057 | -0,005 | -0,009 |
| **Lateral cortex** | **T3D3T** | 0,22 | 0,38 | 0,057 | -0,005 | -0,009 |
| **Lumen** | **C3D4** | 5,00E^-06^ | 0,38 | 0,057 | -0,005 | -0,009 |
|  |  | **Start unloading** | **End unloading** | **Start compression** | **Loading** | **Unloading** |
| **Basal cortex** | **T3D3T** | -0,01 | -0,02 | -0,1 | 0,05 | 0,05 |
| **Lateral cortex** | **T3D3T** | -0,01 | -0,02 | -0,1 | 0,05 | 0,05 |
| **Lumen** | **C3D4** | -0,01 | -0,02 | -0,1 | 0,05 | 0,05 |

*Moreover, the porous-elastic law of behavior governed by the osmotic load can be used to model poroelastic effects. As shown in Figure 6, we integrated this law with the addition of porous elasticity parameters to the volumic elements constituting the cytoplasm of our virtual cells, as listed in Table 4. As described above, these parameters did not lead to better fit of either the individual cell morphology or the global organoid morphology of the synthetic organoid model vs. the biological model. Therefore, we decided not to include them in our modeling approach, and we focused only on the viscoelastic model, to minimize the potential errors.”*

**Incorportation made from the second round of reviewing:**

***From reviewer 1 (R1) questions:***

- Questions 2 and 3 regarding the selection of the values for the different loads used to model the morphological evolution of the organoid, and the possibility to tune them in the poroelastic model to get a better match with the experimental trends.

Our response (italic text above) has been incorporated into the text of the edited manuscript, within the material and method section in which we added the paragraph ‘Optimization of material properties’ from line 619 to line 647. This includes the Table 5 presenting the Range of values used in the preliminary experimental design, and resultant set of parameters applied to our model.

*‘****Optimization of material properties***

*First, we explored the minimum and maximum values allowing for a solution of the system in terms of computation, ensuring that Abaqus could find a convergent solution. The next step involved exploring combinations of these loading values to best represent the morphology observed in the initial stage. After validating the first step of the simulation, which involved inflation of the organoid, we proceeded with the second step of deflation, and finally the last step, which focused on enlargement of the organoid shell. This solution is not unique but is the best that we could achieve in terms of biological relevance for the overall morphology of the organoid. We applied the loads intuitively to simulate the process, on the basis of understanding of the basic mechanisms driving the biological events.*

*To obtain the best possible set of parameters, this problem should ideally be formulated as an inverse problem and solved with numerical optimization algorithms. To set up this inverse problem, we would need time-series data enabling displacement realignment and correlation. However, the non-linearity resulting from the combination of porous elasticity, viscoelasticity, and superelasticity makes this calculation too complex.*

*Tests of arbitrarily chosen combinations of parameters also lead to most non-convergence of the calculation. Because the parameter definition domain is highly irregular, we were unable to perform recalculation of parameters with the conventional optimization method. For illustration, the complete experiment plan with the four parameters at two levels indicated in the table below was conducted. Only six combinations allowed for the convergence of calculation and consequently yielded a usable result. We therefore chose to manually optimize the parameters, guided by the first results of this experiment and approximately ten other complementary trials. The values chosen are derived from manual optimization in the vicinity of the best set of parameters obtained initially (Table 5).*

***Table 5: Range of values used in the preliminary experimental design, and resultant set of parameters applied to our model***

| ***Values*** | ***Bulk modulus*** | ***Poisson ratio*** | ***Tensile limit*** | ***Void ratio*** |
| --- | --- | --- | --- | --- |
| ***Minimum*** | *0.005* | *0.27* | *0.012* | *0.55* |
| ***Maximum*** | *0.02* | *0.35* | *0.017* | *0.65* |
| ***Calculated*** | *0.0209* | *0.3299* | *0.0145* | *0.6* |

*We optimized the model to the best extent possible with the calculated mechanical parameters, but we did not find another solution to the porous elastic problem that produced better results in terms of the global organoid morphology’.*

***From reviewer 3 (R3) questions:***

- Point 16: R3 asked us to emphasize that we add the cell interior resistance, and by combining a continuous approach, we get a full stress tensor.

We added in the manuscript our response to this point (line 456 to line 472):

*‘Thus, our strategy used the 3D VM approach to generate an in silico organoid. To investigate mechanical properties, we incorporated a FEM into this VM. For studying epithelial tissues, VM is widely used, because it represents a set of cells through points connected by segments65. This point-based system serves as the foundation for minimizing an energy function, determining the equilibrium positions of the cell assembly. The 3D positions of the points are decision variables in an optimization problem, which, once solved, provides optimized positions. From these positions, structural and geometrical information, such as cell areas or equilibrium volumes, can be computed.*

*However, whereas the VM provides valuable geometrical information, it lacks the ability to measure mechanical properties. Another challenge, revealed in the AVM application (Fig. 2), arises from the complex elastic response associated with target values of volume, area, or perimeter in AVM66.*

*Our goal is not to invalidate the VM, which serves as a useful representation in configuration space and a first-step model for our approach, but rather to enhance it. By integrating FEM analysis, we can study various stress components, such as plane stress, shear stress, hydrostatic stress, and anisotropic stress associated with deformation.*

*This approach has high potential, because FEM enables refinement of the model's behavior without being hindered by computational complexity.’*
